# Risks and benefits of hypotensive resuscitation in patients with traumatic hemorrhagic shock: a meta-analysis

**DOI:** 10.1186/s13049-018-0572-4

**Published:** 2018-12-17

**Authors:** Natthida Owattanapanich, Kaweesak Chittawatanarat, Thoetphum Benyakorn, Jatuporn Sirikun

**Affiliations:** 1 Division of Trauma Surgery, Department of Surgery, Faculty of Medicine, Siriraj hospital, Mahidol University, 2 Wanglang Road, Bangkok Noi, Bangkok, 10700 Thailand; 2 Department of Surgery, Division of Surgical Critical Care and Trauma, Faculty of Medicine, Chiang Mai University, Chiang Mai, Thailand; 3 Division of Vascular Surgery, Department of Surgery, Faculty of Medicine, Thammasat University, Pathumthani Bangkok, Thailand

**Keywords:** Thailand, Hypotensive resuscitation, Traumatic hemorrhagic shock patients, Meta-analysis

## Abstract

**Background:**

Damage control strategies play an important role in trauma patient management. One such strategy, hypotensive resuscitation, is being increasingly employed. Although several randomized controlled trials have reported its benefits, the mortality benefit of hypotensive resuscitation has not yet been systematically reviewed.

**Objectives:**

To conduct a meta-analysis of the efficacy of hypotensive resuscitation in traumatic hemorrhagic shock patients relative to mortality as the primary outcome, with acute respiratory distress syndrome (ARDS), acute kidney injury (AKI), and multiple organ dysfunction as the secondary outcomes.

**Methods:**

PubMed, Medline-Ovid, Scopus, Science Direct, EMBASE, and CNKI database searches were conducted. An additional search of relevant primary literature and review articles was also performed. Randomized controlled trials and cohort studies reporting the mortality rate associated with hypotensive resuscitation or limited fluid resuscitation were selected. The random-effects model was used to estimate mortality and onset of other complications.

**Results:**

Of 2114 studies, 30 were selected for this meta-analysis. A statistically significant decrease in mortality was observed in the hypotensive resuscitation group (risk ratio [RR]: 0.50; 95% confidence interval [CI]: 0.40–0.61). Heterogeneity was observed in the included literature (I^2^: 27%; degrees of freedom: 23; *p* = 0.11). Less usage of packed red cell transfusions and fluid resuscitations was also demonstrated. No significant difference between groups was observed for AKI; however, a protective effect was observed relative to both multiple organ dysfunction and ARDS.

**Conclusions:**

This meta-analysis revealed significant benefits of hypotensive resuscitation relative to mortality in traumatic hemorrhagic shock patients. It not only reduced the need for blood transfusions and the incidences of ARDS and multiple organ dysfunction, but it caused a non-significant AKI incidence.

## Introduction

Hemorrhagic shock is one of the most common causes of death in trauma or traumatized patients [1]. This is due to the fact that hemorrhagic shock sets in motion a vicious cycle of outcomes, consisting of hypothermia, acidosis, and coagulopathy—otherwise known as the lethal triad. To mitigate these effects, damage control strategies have been proposed, including the early control of bleeding and adequate fluid resuscitation. The aim of hypotensive resuscitation is to maintain systolic blood pressure (or mean arterial pressure) in order to sustain organ perfusion [2, 3]. It was believed that induced intraoperative hypotension could lead to reduced blood loss and fewer transfusions [4–6]. However, much recent data have shown that there is a significant relationship between hypotensive resuscitation and postoperative renal injuries [7–9].

Fluid resuscitation with the rapid administration of intravenous fluids until the blood pressure is normalized is the traditional fluid-resuscitation strategy. A number of randomized controlled trials have demonstrated some improvement in survival and mortality using a liberal fluid hypotensive resuscitation. Nevertheless, other studies have concluded that a more conservative hypotensive strategy—in which minimal amounts of fluids are given until the severe bleeding has been controlled—is a more efficacious resuscitation strategy [10–13]. Although the volume of data on hypotensive resuscitation continues to grow, conflicting findings are being reported regarding the efficacy of this trauma-mitigating strategy. Accordingly, the aim of this study was to conduct a meta-analysis of the efficacy and drawbacks of hypotensive resuscitation in traumatic hemorrhagic shock patients relative to mortality as the primary outcome, with acute respiratory distress syndrome, acute kidney injury, and multiple organ dysfunction as the secondary outcomes.

## Methods

### Inclusion criteria

This meta-analysis included only randomized controlled trials (RCTs) and cohort studies that evaluated adult patients aged older than 18 years who had traumatic hemorrhagic shock and a systolic blood pressure below 90 mmHg.

The intervention assessed was conventional fluid resuscitation with normotension (liberal fluid resuscitation) versus hypotensive resuscitation (limited fluid resuscitation). Conventional fluid resuscitation was defined as liberal fluid resuscitation until the systolic blood pressure exceeded 90 mmHg (normal blood pressure). Hypotensive resuscitation was defined as limited fluid resuscitation to maintain adequate organ perfusion, with a systolic blood pressure of ~ 70–80 mmHg or a mean arterial pressure of ~ 50 mmHg.

Excluded were studies of patients who were pregnant or had traumatic brain injuries, research with insufficient mortality data, and investigations that had not received ethical approval.

### Outcomes assessed

The primary outcome was all-cause mortality, as reported by the authors of the included studies. The secondary outcomes included the rates of the following morbidities: acute respiratory distress syndrome (ARDS), acute kidney injury (AKI), and multiple organ dysfunction (MODS). Other secondary outcomes included the fluid resuscitation volume and the transfusion of packed red cells.

### Search methods

PubMed, Medline-Ovid, Scopus, Science Direct, EMBASE, and CNKI database searches were conducted for articles published before January 31, 2018. An additional search of relevant primary literature and review articles was also performed. The references from identified studies that appeared to be germane to the topic of study were hand-searched. The medical subject headings (MeSHs) used in our searches included “hypotensive resuscitation” or “limited fluid resuscitation” and “trauma” or “trauma*”. There was no language restriction.

### Data collection

Two reviewers (N.O. and T.B.) independently inspected each article identified during the search, scanned the full text of relevant articles, applied the inclusion and exclusion criteria, and extracted and recorded the data. Disagreements relating to any aspect of the data extraction process were resolved by discussion with a third reviewer (K.C.), with the final decision made by consensus.

### Quality assessment

The quality of included studies was evaluated using the Jadad quality assessment scale for randomized controlled studies. Our subsequent analysis only included those studies that scored ≥2 on the scale (which indicated that their results were valid) [14]. To assess the quality of nonrandomized studies, the Newcastle–Ottawa Scale (NOS) was used. This 3-item scoring system evaluates the selection of the participants; the comparability between the groups; and the ascertainment of exposure for case-control studies, and the outcome of interest in the case of cohort studies [15].

### Statistical analysis

All statistical analyses were performed using Review Manager 5.3 software from the Cochrane Collaboration (London, United Kingdom). We extracted the proportions and 95% confidence intervals from each study and pooled them using the random effects model. Cochran’s Q test was performed and quantified using the *I*^*2*^ statistic to evaluate the statistical heterogeneity among the included studies. The *I*^2^-values were categorized as follows: 0–25% indicated insignificant heterogeneity; 26–50%, low heterogeneity; > 50% to ≤75%, moderate heterogeneity; and > 75%, high heterogeneity [16]. The fixed effects model was used for analyses that evaluated data with no significant heterogeneity. Funnel plots were used to evaluate for publication bias. *P*-values less than 0.05 were considered statistically significant.

## Results

### Search results and study characteristics

**Fig. 1:**
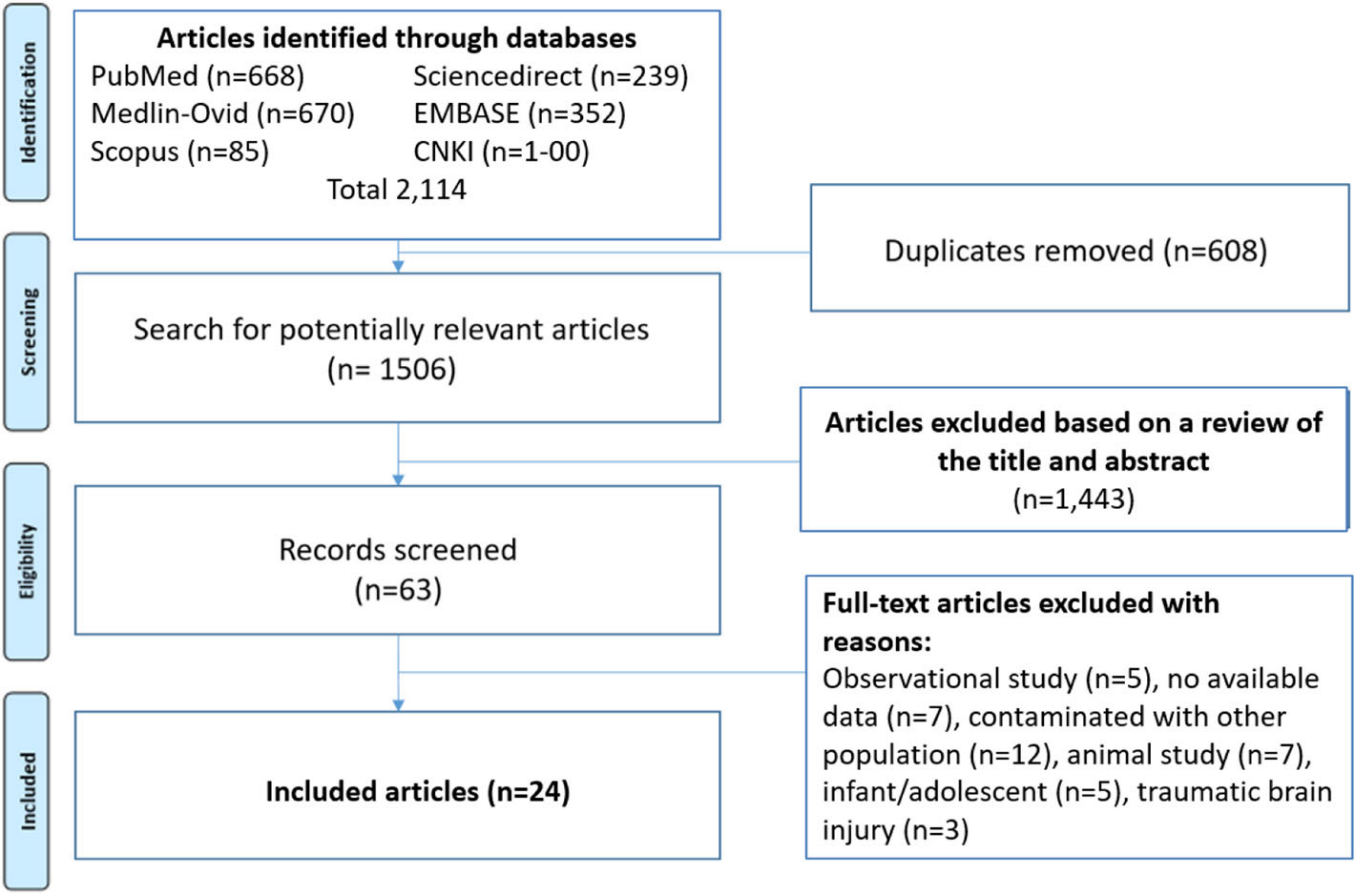
Flow diagram of the article selection procedure based on the Preferred Reporting Items for Systematic Reviews and Meta-Analyses (PRISMA) guideline

Figure 1 illustrates a flow diagram describing the article selection process, which was based on the Preferred Reporting Items for Systematic Reviews and Meta-Analyses (PRISMA) guidelines. Our review of the literature yielded 2114 publications up to January 31, 2018. For an assortment of reasons during the article selection process, 2090 of those articles were excluded. The specific reasons for exclusion are given in Fig. 1. The remaining 24 studies were included in the final analysis [2, 10–13, 17–35]. Of those 24 studies, 20 were randomized controlled trials, while 4 were prospective cohort studies. The baseline characteristics described in Table 1 comprise country, article type, the quality assessment score for each study, and the intervention definition.

**Table 1:** Study characteristics

Study	Country	Study design	Jadad Quality score/Newcastle-Ottawa scale (NOS)	Participants	Intervention	Control
Bickell 1994	USA	Single-center, prospective RCT	Three out of five -No double blind	Gunshot or stab wounds to the torso who had SBP<90 mmHg	Delayed resuscitation with RLS 10ml/hr until definitive treatment	Immediate resuscitation to maintain SBP at least 100 mmHg
Dutton 2002	USA	Single-center, prospective RCT	Three out of five -No double blind	Traumatic hemorrhagic shock with SBP <90 mmHg and evidence of ongoing bleeding	Low SBP of 70 mmHg	Conventional SBP > 100 mmHg
WANG Mei-tang 2007	China	Single-center, prospective cohort study	Selection: 3 Comparability: 2 Outcome: 2	Traumatic hemorrhagic shock	Preoperative SBP approximately 70-80 mmHg	Preoperative SBP >90 mmHg
ZHENG Wei-hua 2007	China	Single-center, prospective, RCT	Two out of five -No method of randomization -No double blind	Traumatic hemorrhagic shock patients	Limited fluid resuscitation (MAP 50-60 mmHg)	Aggressive fluid resuscitation (MAP 70 mmHg)
HUA Li-dain 2010	China	Prospective, RCT	Two out of five -No method of randomization -No double blind	Severe multiple hemorrhagic shock	Limited fluid resuscitation (SBP 70 mmHg)	Observational with MAP at least 90/60 mmHg
WANG Aitian 2010	China	Prospective, RCT	Two out of five -No method of randomization -No double blind	Traumatic hemorrhagic shock	Limited fluid resuscitation to maintain SBP 70 mmHg	Conventional resuscitation to maintain SBP 100 mmHg
Fan Hai-Peng 2011	China	Prospective, RCT	Two out of five -No method of randomization -No double blind	Pelvic fracture with hemorrhagic shock	Low MAP 50-60 mmHg or SBP 70-90 mmHg	Conventional MAP 60-80 mmHg or SBP >100 mmHg
Morrison 2011	China	Single-center, prospective, two-arm, intention to treat, RCT	Three out of five -No double blind	Patients undergoing laparotomy or thoracotomy for blunt and penetrating trauma who had SBP < 9o mmHg	Experimental group with MAP 50 mmHg	Control group with MAP 65 mmHg
Fan Hai-Peng 2012	China	Single-center, RCT	Two out of five -No method of randomization -No double blind	Hepatic and splenic injury with hemorrhagic shock	Limited fluid resuscitation (MAP 50-60 mmHg)	Conventional resuscitation (SBP 100 mmHg or MAP 60-80 mmHg)
LI Wenhao 2012	China	Prospective, RCT	Two out of five -No method of randomization -No double blind	Traumatic hemorrhagic shock without controlling bleeding	Limited fluid resuscitation (MAP 55 mmHg)	Adequate fluid resuscitation (MAP 75mm Hg)
Chen Mu-hu 2013	China	Prospective, RCT	Two out of five -No method of randomization -No double blind	Traumatic hemorrhagic shock patients	Limited fluid resuscitation (SBP 70 mmHg)	Aggressive fluid resuscitation (SBP 90 mmHg)
ZHAO yong-gang 2013	China	Retrospective cohort study	Selection: 4 Comparability: 2 Outcome: 2	Traumatic hemorrhagic shock patients	Objective group (SBP 85 mmHg, limited fluid)	Control group (SBP >90 mmHg, rapid and full replenishment of fluid
WANG Xiao-guo 2014	China	Prospective, RCT	Two out of five -No method of randomization -No double blind	Traumatic liver and splenic injury	Limited fluid resuscitation (MAP 50-70 mmHg)	Conventional resuscitation (MAP 70-90 mmH)
ZENG Fan-yuan 2014	China	Single-center, cohort study	Selection: 4 Comparability: 2 Outcome: 2	Uncontrolled traumatic hemorrhagic shock patients	Experimental group (MAP 50 mmHg)	Control group (MAP 70 mmHg)
Chen Mianzhan 2015	China	Prospective, RCT	Two out of five -No method of randomization -No double blind	Traumatic hemorrhagic shock patients	Limited resuscitation (SBP at least 80 mmHg)	Conventional resuscitation (SBP at least 90 mmHg)
Chen Yuan-bing, 2015	China	Prospective, RCT	Two out of five -No method of randomization -No double blind	Traumatic hemorrhagic shock patients	Limited resuscitation (SBP 70 mmHg)	Conventional resuscitation (SBP >90 mmHg)
Huang Ting 2015	China	Prospective, RCT	Two out of five -No method of randomization -No double blind	Traumatic hemorrhagic shock	Control group with MAP 40-60 mmHg	Observation group with MAP 60-90 mmHg
Schreiber 2015	USA	Multi-center, RCT	Three out of five -No double blind	Blunt or penetrating trauma patients with SBP <90 mmHg	Administer 250 ml of fluid if SBP <70 mmHg or absent radial pulse	Administer 2 liters initially and additional fluid as needed to maintain SBP > 110 mmHg
Wen Zhen-jie 2015	China	Multi-center, prospective cohort studies	Selection: 4 Comparability: 2 Outcome: 2	Traumatic hemorrhagic shock	Limited fluid resuscitation (SBP 75 mmHg)	Conventional fluid resuscitation (SBP > 100 mmHg)
XU Guoping 2015	China	Single center, prospective RCT	Two out of five -No method of randomization -No double blind	Traumatic hemorrhagic shock patients	Limited fluid resuscitation (MAP 40-60 mmHg or SBP 70 mmHg)	Conventional resuscitation (MAP 60-80 mmHg or SBP > 90 mmHg)
YAO Jian-hui 2015	China	Single center, prospective, RCT	Two out of five -No method of randomization -No double blind	Multiple traumatic hemorrhagic shock patients	Limited fluid resuscitation (MAP 40-50 mmHg)	Active fluid resuscitation (MAP 60-80 mmHg)
Carrick 2016	USA	Single-center, prospective, two-arm, intention-to-treat, RCT	Three out of five -No double blind	Penetrating trauma patients with SBP < 90 mmHg who were brought emergently to OR for bleeding control	Keep low MAP (MAP 50 mmHg)	Keep normotension (MAP at least 65 mmHg)
Dai Yulong, 2016	China	Prospective, RCT	Two out of five -No method of randomization -No double blind	Traumatic hemorrhagic shock patients	Limited fluid resuscitation (SBP 65 mmHg)	Conventional resuscitation (SBP 90 mmHg)
Wang Fengyong 2016	China	Single center, prospective RCT	Two out of five -No method of randomization -No double blind	Active hemorrhagic shock	Limited fluid resuscitation (maintain MAP 40-60 mmHg)	Conventional resuscitation (maintain MAP 60-90 mmHg)

### Assessment of reporting bias

**Fig. 2:**
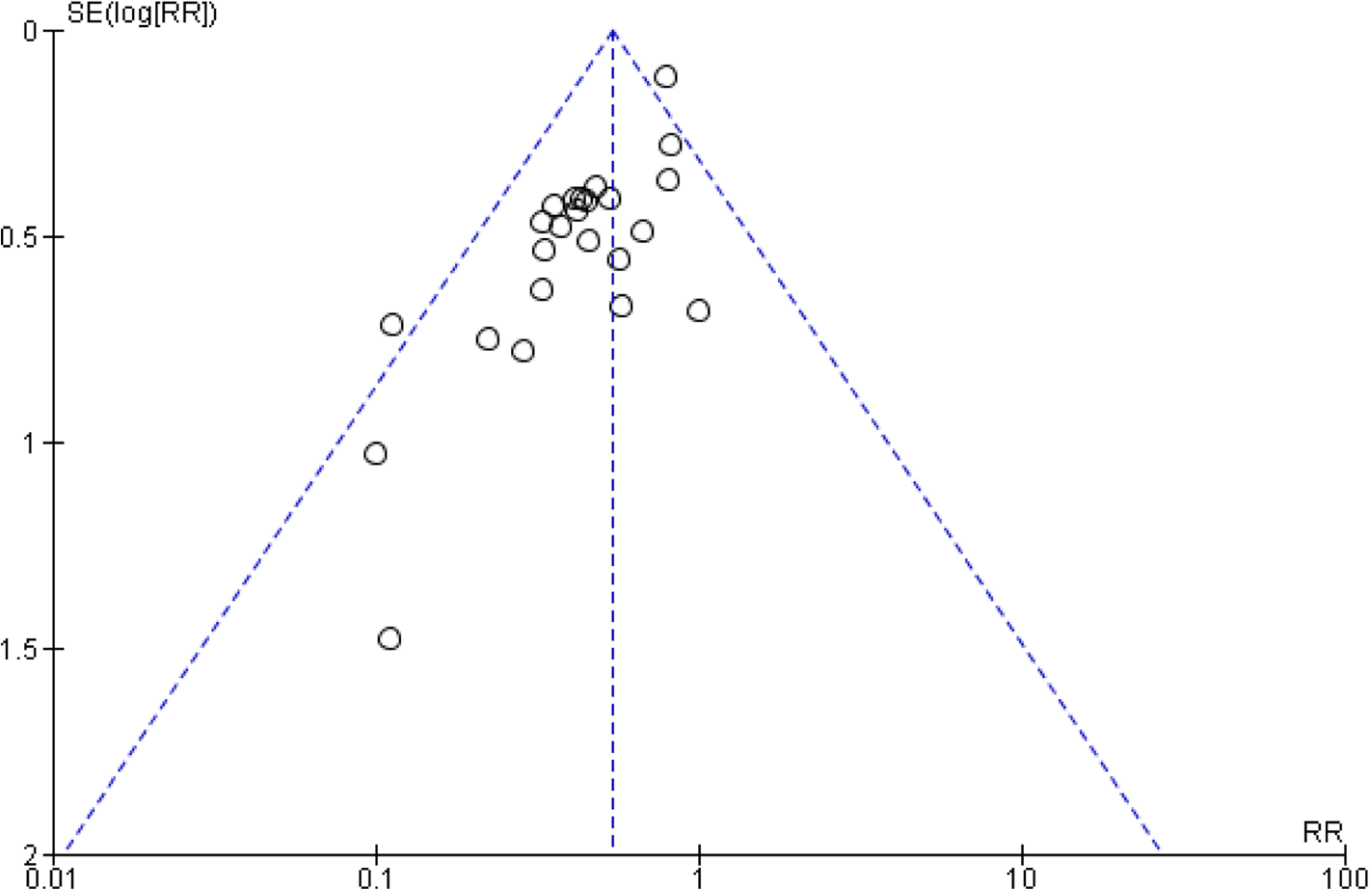
Funnel plot of reporting bias (the dotted lines indicate the 95% confidence interval [CI]; SE, standard error; RR, risk ratio)

A funnel plot of the reporting bias shows asymmetry on visual inspection (Fig. 2). There were 3 studies with low statistically significant effects (risk ratio [RR]: 0.11; 95% confidence interval [CI]: 0.01–0.75; Zhao Yong-gang, 2013 [34], Dai Yulong, 2016 [20], and Wang Fengyong, 2016 [27]). These studies give the plot an asymmetrical appearance, with a gap at a bottom corner of the graph. In this setting, the effect calculated in a meta-analysis will tend to overestimate the intervention effect. However, after reviewing the study design and evaluating the outcomes, we still decided to include them in our analysis.

**Fig. 3:**
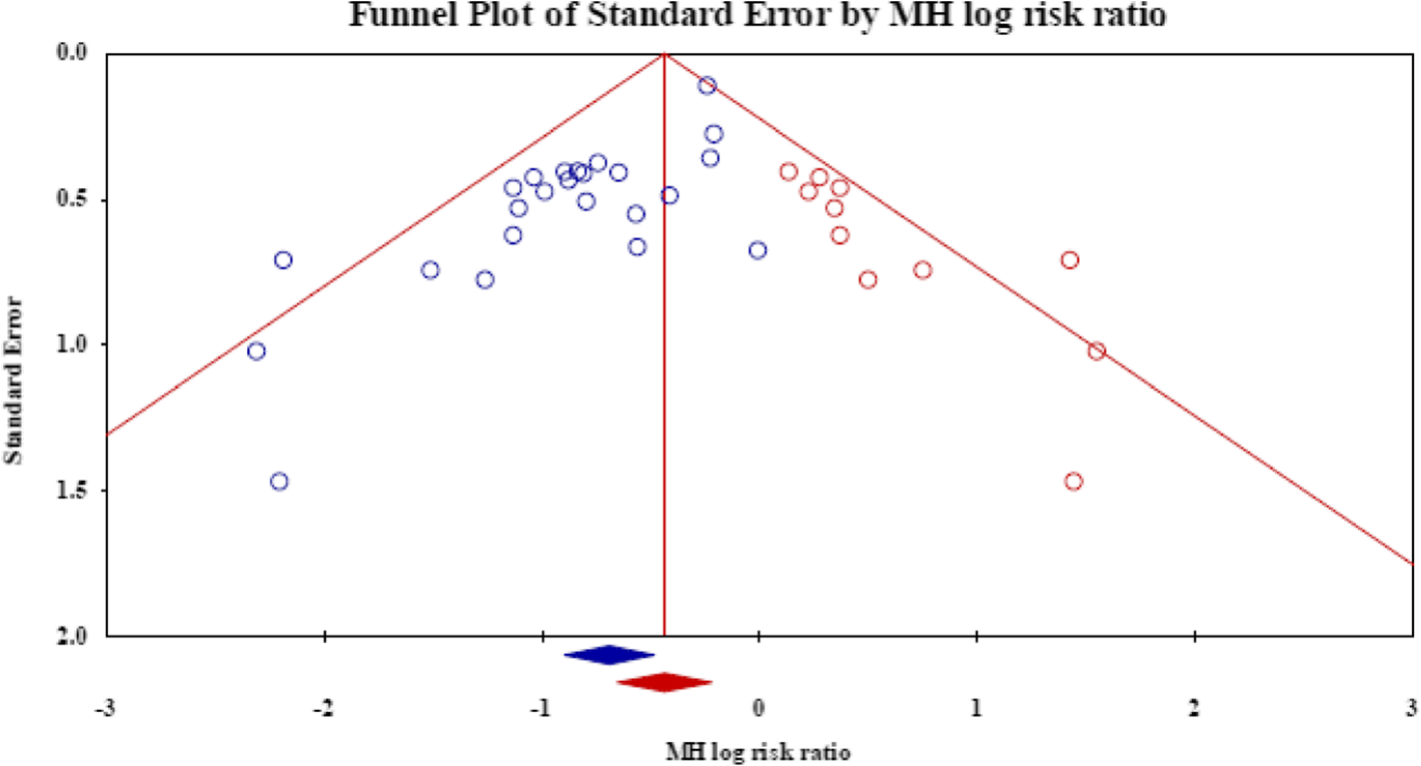
The funnel plot after trim and fill method

The Egger test found a strong significance in publication bias (*P* = 0.000) Therefore, the trim and fill method was conducted to adjust the publication bias by removing the smaller studies causing asymmetry and replacing the omitted studies. This method has been claimed to improve the effect size and confidence intervals [36]. As a result, the present study drew upon 11 hypothesized studies. The new RR was 0.65 and CI (0.52–0.80), which showed significant difference in mortality benefit (Fig. 3).

### Synthesis of primary outcome

**Fig. 4:**
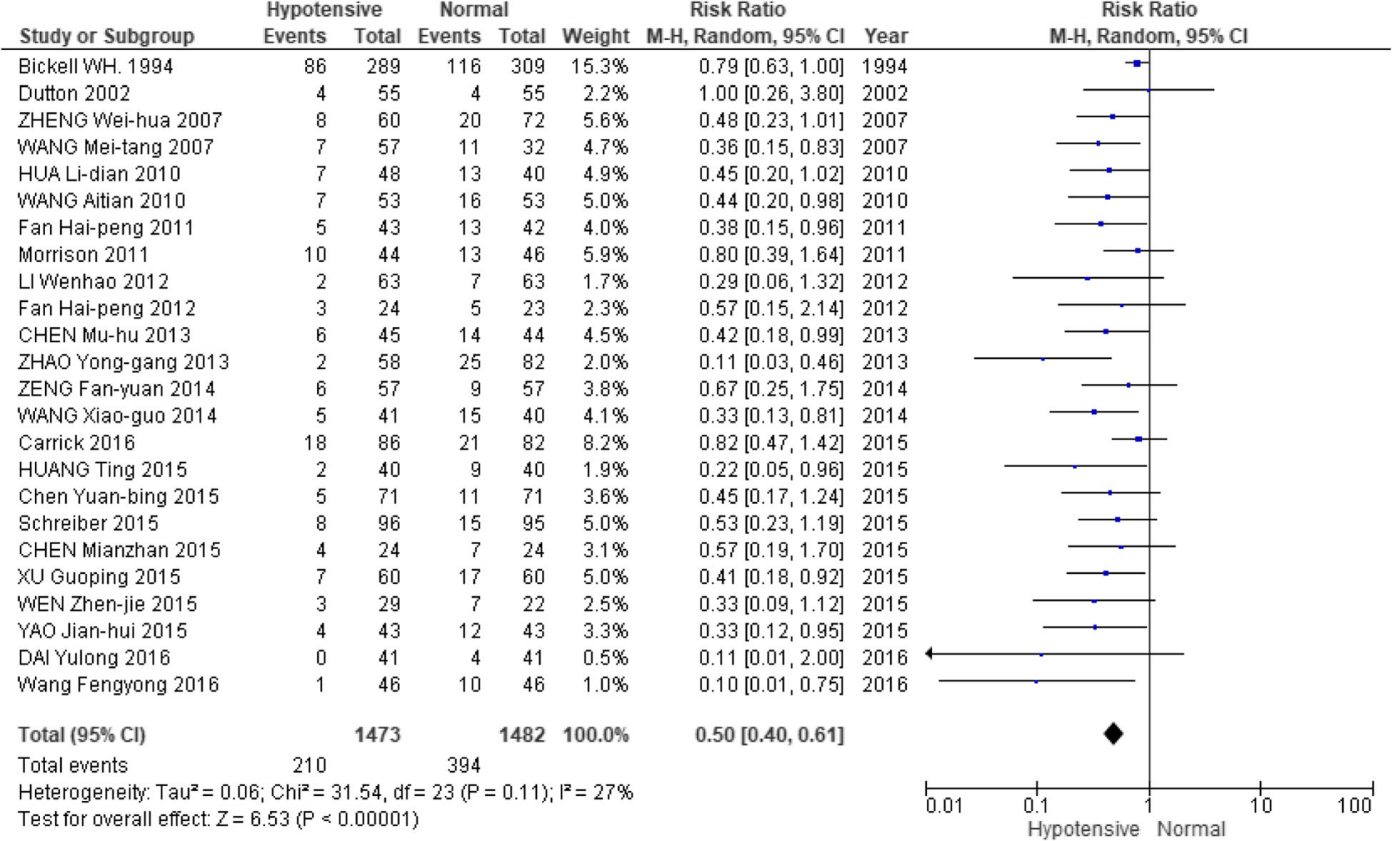
Forest plot of association between hypotensive resuscitation and normal resuscitation, relative to mortality

A pooled analysis was performed of the 24 studies using a random-effects model, with findings reported as RR and 95% CI (*n* = 1473; RR: 0.50; 95% CI: 0.40–0.61). A mild heterogeneity among these 24 studies was observed (Q test: 0.11, which is greater than 0.1; I^2^: 27%). Given the degree of homogeneity among the studies, a random-effects model was subsequently applied (Fig. 4). As described in Fig. 4, some overtime bias was observed among the studies. Although the first published study on this topic (by Bickell WH et al. [2]) was published in 1994, we still included it due to its sound methodology and overall high level of quality.

### Secondary outcomes

**Fig. 5:**
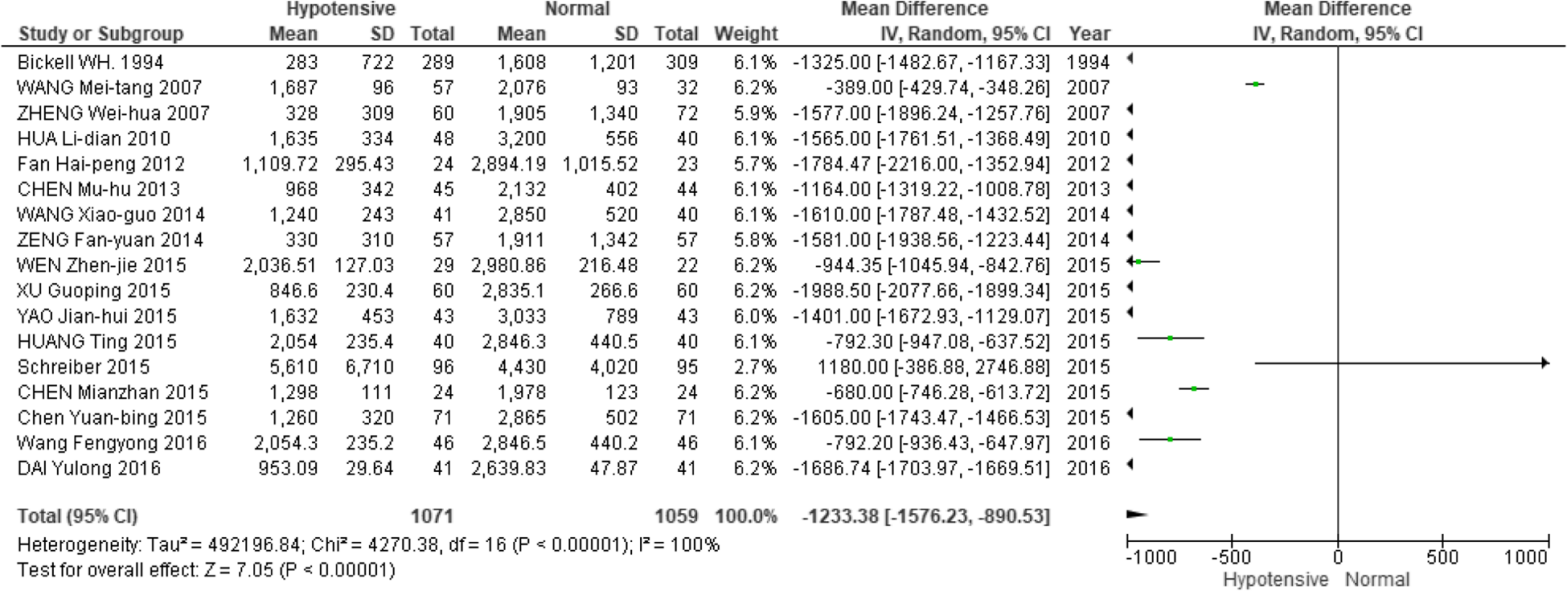
Forest plot of association between hypotensive resuscitation and normal resuscitation, relative to fluid resuscitation volume

**Fig. 6:**
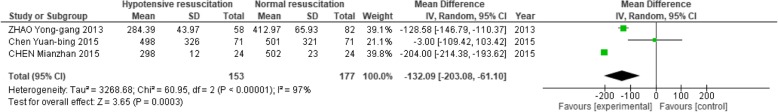
Forest plot of association between hypotensive resuscitation and normal resuscitation, relative to transfusion of packed red cells

The hypotensive resuscitation group had a lower amount of fluid resuscitation and packed red cell transfusion than the normotensive resuscitation group (Figs. 5 and 6). The mean difference in fluid resuscitation between groups was 1233 ml (95% CI: -1576, -890) and red cell transfusion was 132 ml (95% CI: -203, -61).

**Fig. 7:**
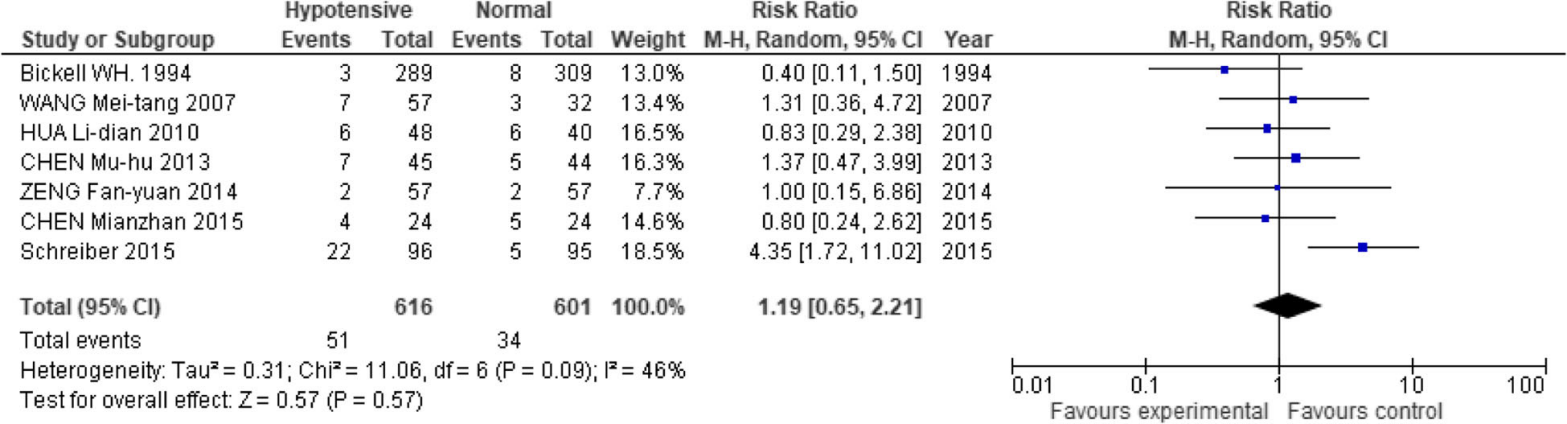
Forest plot of association between hypotensive resuscitation and normal resuscitation, relative to acute kidney injury (AKI)

**Fig. 8:**
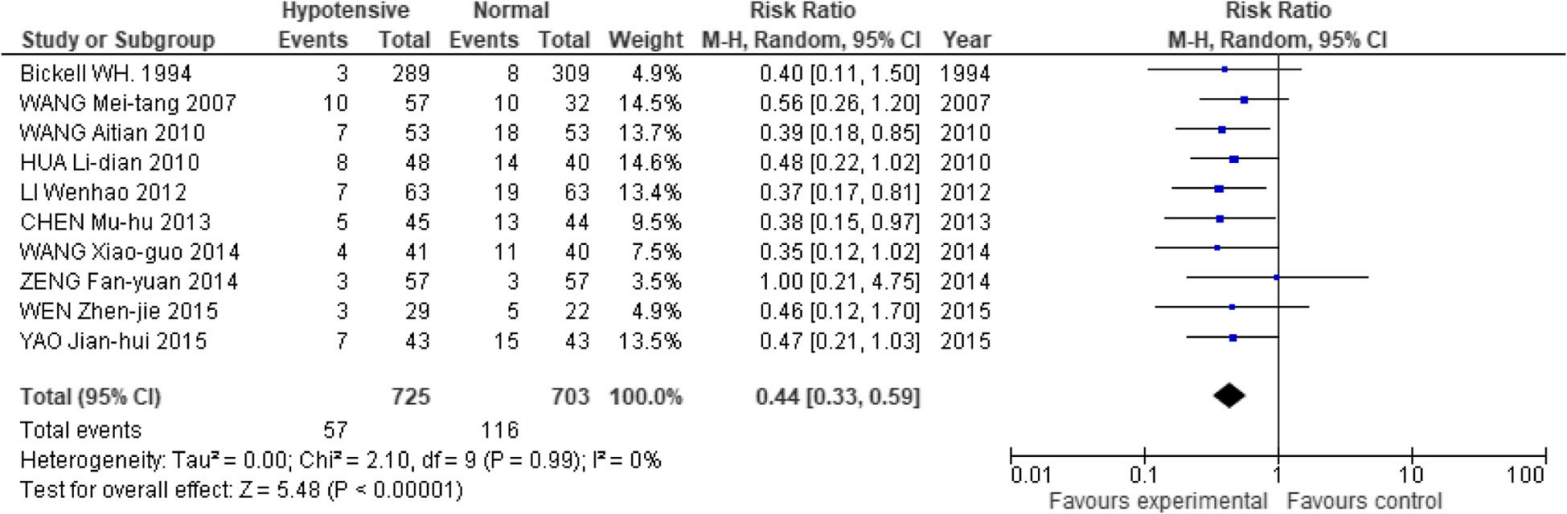
Forest plot of association between hypotensive resuscitation and normal resuscitation, relative to acute respiratory distress syndrome

**Fig. 9:**
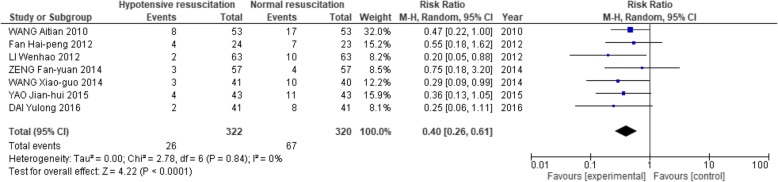
Forest plot of association between hypotensive resuscitation and normal resuscitation, relative to multiple organ dysfunction

In terms of the possible risks of this hypotensive strategy, 7 studies [2, 13, 17, 18, 23, 28, 33] showed that the hypotensive resuscitation group had no significant difference in acute kidney injury (RR: 1.19; 95% CI: 0.65, 2.21; Fig. 7). This strategy also had lower incidences of both acute respiratory distress syndrome (RR: 0.44; 95% CI: 0.33, 0.59) and multiple organ dysfunction (RR: 0.40; 95% CI: 0.26, 0.61; Figs. 8 and 9).

## Discussion

During the last decade, two differing approaches to damage control have been used for severe trauma patients. The traditional liberal fluid approach has, however, come under increasing attention due to the possible complications associated with administering more than 2 l of fluid before surgery. In contrast, hypotensive resuscitation is being increasingly employed because many animal studies have found its use is associated with reduced effects of the lethal triad. The concept of this strategy is to restrict the amount of fluid resuscitation to maintain a low enough blood pressure for adequate cerebral perfusion. To avoid further bleeding due to dilution coagulopathy and to dislodge hemostatic blood clots, hypotensive resuscitation has been adopted as a part of damage control resuscitation for trauma patients [37, 38]. Bickell WH et al. were the first to study and publish the role of hypotensive resuscitation in penetrating trauma patients. They showed that hypotensive resuscitation before surgery could significantly decrease mortality. Not only did the fatality rate decrease, but the hypotensive resuscitation also appeared to correlate with less coagulopathy and other complications, including less cardiovascular failure, respiratory failure, and acute kidney injury (AKI).

Despite the sizeable number of studies on hypotensive resuscitation in animal models and in humans, there is only one Cochrane-based review, and that review pertains only to the timing and volume of fluid administration in patients with bleeding. Moreover, in the present study, all types of hemorrhagic shock were included, including gastrointestinal bleeding. The results of this meta-analysis showed that there is no evidence for or against an early or larger volume of intravenous fluid administration in cases of uncontrolled hemorrhage.

Given that there is no meta-analysis related to studies comparing hypotensive to liberal fluid resuscitation, we decided to perform such an investigation. It is noteworthy that a large majority of the present literature had to be excluded from further analysis. This was mainly due to either the presence of irrelevant data and/or the lack of sufficient data. This highlights and supports the validity of the Cochrane approach.

Our analysis revealed that hypotensive resuscitation (i.e., limited fluid resuscitation) has beneficial effects on survival in traumatic hemorrhagic shock. Even in the blood product based resuscitation era, all the literature still shows significant survival benefits [10–13]. Hypotensive resuscitation is also associated with a significantly lower amount of fluid resuscitation and packed red cell transfusion, and a significantly lower incidence of acute respiratory distress syndrome and multiple organ dysfunction. In contrast, no significant difference was observed between resuscitation methods relative to the incidence of acute kidney injury. Moreover, we were not able to identify any report of renal replacement therapy and/or long-term dialysis in traumatic hemorrhagic shock patients.

The subgroup analysis that we performed showed a mortality benefit of hypotensive resuscitation in traumatic hemorrhagic shock with coexisting traumatic brain injury. This might alert readers to carefully interpret the reported findings from Gong Hong-chang et al. and LIU Wei-zheng et al., whose studies both maintained low normal blood pressure (SBP 90 mmHg or MAP 60–70 mmHg). Meanwhile, LIU Yu et al. maintained SBP 70 mmHg, which truly met the definition of hypotensive resuscitation. However, the data is not compelling enough to strongly recommend hypotensive resuscitation in traumatic hemorrhagic shock patients with traumatic brain injury, and there were no reported data on the functional outcomes after this strategy.

This study supports the use of hypotensive resuscitation, not only in penetrating wound patients, but also in other types of traumatic injuries. This significant finding was based on low heterogeneity. However, the limitations of this analysis include the fact that some clinical and methodological heterogeneities between the studies cannot be ruled out, and there may be some overtime bias. Despite significant publication bias shown by an asymmetrical funnel plot and Egger test, the trim and fill method also showed a statistically significant mortality benefit from the hypotensive strategy. Although a large, multi-center, randomized controlled trial should be conducted, the likelihood of such an approach succeeding given the present literature (including the present review) seems unlikely.

## Conclusions

The results of this meta-analysis revealed a significant benefit of hypotensive resuscitation relative to mortality in traumatic hemorrhagic shock patients. Moreover, hypotensive resuscitation was found to be a significantly more effective strategy than traditional fluid resuscitation in terms of acute respiratory distress syndrome and multiple organ dysfunction.
